# Decline in Kelp in West Europe and Climate

**DOI:** 10.1371/journal.pone.0066044

**Published:** 2013-06-26

**Authors:** Virginie Raybaud, Grégory Beaugrand, Eric Goberville, Gaspard Delebecq, Christophe Destombe, Myriam Valero, Dominique Davoult, Pascal Morin, François Gevaert

**Affiliations:** 1 Université Lille 1, UMR 8187 LOG, Laboratoire d’Océanologie et de Géosciences, Wimereux, France; 2 CNRS, UMR 8187 LOG, Laboratoire d’Océanologie et de Géosciences, Wimereux, France; 3 SAHFOS, Plymouth, United Kingdom; 4 Université Lille 1, UMR 8198 GEPV, Laboratoire de Génétique et Evolution des Populations Végétales, Villeneuve d’Ascq, France; 5 UPMC, UMR 7144 AD2M, Laboratoire « Adaptation et diversité en milieu marin », Station biologique de Roscoff, Roscoff, France; 6 CNRS, UMR 7144 AD2M, Laboratoire « Adaptation et diversité en milieu marin », Station biologique de Roscoff, Roscoff, France; National Institute of Water & Atmospheric Research, New Zealand

## Abstract

Kelp ecosystems form widespread underwater forests playing a major role in structuring the biodiversity at a regional scale. Some seaweeds such as *Laminaria digitata* are also economically important, being exploited for their alginate and iodine content. Although some studies have shown that kelp ecosystems are regressing and that multiple causes are likely to be at the origin of the disappearance of certain populations, the extent to which global climate change may play a role remains speculative. Here we show that many populations of *L. digitata* along European coasts are on the verge of local extinction due to a climate-caused increase in sea temperature. By modeling the spatial distribution of the seaweed, we evaluate the possible implications of global climate change for the geographical patterns of the species using temperature data from the Coupled Model Intercomparison Project phase 5 (CMIP5). Projections of the future range of *L. digitata* throughout the 21st century show large shifts in the suitable habitat of the kelp and a northward retreat of the southern limit of its current geographic distribution from France to Danish coasts and the southern regions of the United Kingdom. However, these projections depend on the intensity of warming. A medium to high warming is expected to lead to the extirpation of the species as early as the first half of the 21st century and there is high confidence that regional extinction will spread northwards by the end of this century. These changes are likely to cause the decline of species whose life cycle is closely dependent upon *L. digitata* and lead to the establishment of new ecosystems with lower ecological and economic values.

## Introduction

Anthropogenic climate change is already affecting biological and ecological systems worldwide and several kinds of responses such as phenological, biogeographical and abrupt ecosystem shifts have been documented [1], [2]. Kelp forests, highly productive and emblematic ecosystems of both cold-temperate and polar coastal regions [3], may be substantially affected by global warming [4], [5]. The species *Laminaria digitata* is among the most commercially important species of European kelps and some populations have declined for the past few years [6]. Since the 19^th^ century, they have been exploited by the iodine industry and more recently they have been harvested for their content in alginate, which is used as a binder, emulsifier and as a molding material in a broad range of products [7].

The distribution of *L. digitata* on the European coasts ranges from the southern coasts of Brittany (Quiberon bay) to the Northern part of the Norway [8], [9]. The macroalga is absent from the Baltic Sea and is sparsely distributed on the southeastern coasts of Brittany. It is found in the upper sub-littoral fringe in sheltered or moderately exposed sites and exclusively on rocky shore. It supports high hydrodynamics and prefers clear or slightly turbid waters. The temperature is a primordial factor regulating the horizontal extension of *L. digitata* [6], [10]. Its thermal optimum ranges from 10 to 15°C, reproduction is impaired beyond 18°C [11] and death occurs when temperature reaches 22°C [12], [13]. Bolton and Lüning [14] found a value between 22°C and 23°C (with cell damages at 23°C). The vertical extent of this species varies according to light penetration and is therefore inversely related to turbidity. In general, the macroalga develops between +1 and −3 m but can be exceptionally found down to 20 m in very clear waters [11]. The main competitor of *L. digitata* is the species *Sacchoriza polyschides* [15]. This annual species grows more rapidly than *L. digitata* and usually colonize spaces previously occupied by *L. digitata.*

The decline in the abundance of *L. digitata* along the European coasts have gained attention recently. This phenomenon has been observed in Brittany [7], in Normandy [16] and in the English Channel [17]. Although exploitation may have contributed to the decline of the species, the implication of global warming is possible. To examine the potential influence of global climate change on *L. digitata*, we modeled the ecological niche (*sensu* Hutchinson, [18]) of the macroalga and projected its potential distribution along the European coasts for the 21^st^ century. Such large-scale projections of changes in this marine forest canopy have never been addressed despite the fact that degradation of these habitats may have considerable ecological and economic consequences. To our knowledge, only three previous studies have applied Ecological Niche Models (ENM) on kelps: a predictive model of subtidal kelp forest in Brittany-France [19] and two spatial predictive distribution models of the kelp species *Laminaria hyperborea* [20] and *Saccharina latissima* [21] in Norway. All of them used Generalized Additive Models on a small area and did not investigate the effects of climate change on the whole species distribution.

In the present study, we apply a new ENM based on presence-only data and called Non-Parametric Probabilistic Ecological Niche model (NPPEN). This model offers many advantages [22]: (*i*) it requires presence-only data, (*ii*) it is based on a non-parametric test and is therefore distribution-free, (*iii*) by the use of the generalized Mahalanobis distance, the model takes into account the correlation between environmental variables. As for any ENM, this method has assumptions and limitations, which will be later discussed.

This model, recently applied to marine fish [22], [23] and the macro-benthic fauna [24], is used to identify the ecological niche (*sensu* Hutchinson, [18] ) of *L. digitata* and to model its current spatial distribution, taken as reference the period 1982–2009. We then evaluate the possible implications of global climate change for the geographical patterns of the species in the 21^st^ century using climatic scenarios from the Coupled Model Intercomparison Project phase 5 (CMIP5, [25], [26]). These scenarios, called ‘Representative Concentration Pathways’ (RCP), are the latest generation of earth system models designed in the framework of the Fifth Assessment Report by the Intergovernmental Panel on Climate Change (IPCC AR5). Four greenhouse gas concentration trajectories were chosen by the IPCC: RCP2.6, RCP4.5, RCP6.0 and RCP8.5 (from the most optimistic to the most pessimistic). They are labeled according to their total radiative forcing in 2100 [25], [26].

## Materials and Methods

### Occurrence Data of *Laminaria digitata*

Because no comprehensive database of the distribution of *L. digitata* existed, we compiled occurrence data from different sources to optimize our assessment of the recent distribution of this species. We used data from OBIS (http://www.iobis.org/) and the Ecokelp project (http://www.sb-roscoff.fr/ecokelp/images/marine/ecokelp-36month-report-fin.pdf). We completed these databases with information found in the literature [8], [9], [12], [27], [28], [29], [30]. Distribution map of occurrence records is presented in Fig. S1.

### Large-scale Abiotic Parameters

Monthly mean gridded Sea Surface Temperature (SST) data from 1982 to 2009 were taken from NOAA 4 km Advanced Very High Resolution Radiometer (AVHRR) Pathfinder [31] (http://www.nodc.noaa.gov/SatelliteData/pathfinder4km/). Maximum annual SST was calculated for the Northern European region (45°N to 71.5°N; 11.5°W to 27.0°E). Bathymetric data were obtained from the “Smith and Sandwell Global Seafloor topography” [32] (http://iridl.ldeo.columbia.edu/SOURCES/.Sandwell/.seafloor/; NOAA and Scripps Institution of Oceanography). Average Sea Surface Salinity (SSS) data were taken from the Levitus’ climatology [33]. ICES data (http://www.ices.dk) were used to complete this database in coastal regions where there is no assessment of annual SSS (e.g. some regions of the eastern English Channel). Data on substratum types were obtained from SHOM database (Service Hydrographique et Océanographique de la Marine). The diffuse attenuation coefficient for downwards irradiance at 490 nm (Kd490) was used as an indicator of the turbidity. Climatological data were downloaded from NASA’s Giovanni portal [34] (http://gdata1.sci.gsfc.nasa.gov/daac-bin/G3/gui.cgi?instance_id=ocean_month).

### Climate Scenarios

We used the new “Representative Concentration Pathways” (RCP) climate scenarios, which will be a part of the fifth Intergovernmental Panel on Climate Change (IPCC) assessment report [25] (http://cmip-pcmdi.llnl.gov/cmip5/data_portal.html). The outputs (2006–2100) of simulated SST from two high resolution climate models were used in this study (CNRM-CM5 and MPI-ESM-LR), with the following available RCP scenarios: the low RCP2.6, the medium RCP4.5 and the high RCP8.5.

### Modeling Procedure

#### Modeling of the ecological niche of *L. digitata*

We applied the Non-Parametric Probabilistic Ecological Niche model (NPPEN) to estimate the niche of *L. digitata* [22]. NPPEN is a new numerical tool that allows the modeling of a species ecological niche and the mapping of its spatial distribution, by calculating its probabilities of occurrence. In contrast to many ecological niche models, this method only requires presence data. As NPPEN is fully described elsewhere [22], we will only briefly recall the main steps of calculation. The first step consists in constructing a reference matrix (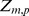) of the environmental data corresponding to the presence records. The matrix is therefore homogenized to *(i)* eliminate the potential effect of over-sampling and *(ii)* remove as far as possible the inaccurate reporting of occurrence. In a second step, the Mahalanobis generalized distance is calculated between the observations and the homogenized reference matrix using the following formula:



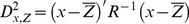
(1)With *x* the vector of length *p*, representing the values of the environmental data to be tested, *R_p,p_* the correlation matrix of reference matrix 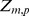 and 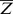 the average environmental conditions inferred from 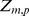. The use of the Mahalanobis distance instead of a classical Euclidian distance presents a double advantage: it enables the correlation between variables to be taken into account [35] and is independent of the scales of the descriptors [36]. In the third step, the model calculates the probability of each grid point to belong to the reference matrix by using a simplified version of the Multiple Response Permutation Procedure (MRPP) [37]. This probability (ν) is the number of times the simulated distance was found greater or equal than the observed average distance:



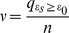
(2)With *ε_o_* is the average observed distance, *ε_s_* the recalculated distance after permutation and *n* the maximum number of permutations. If the probability is close to 1, the environmental value of the tested point are at the centre of the ecological niche. A probability close to zero indicates that the environmental conditions of the point are outside of the ecological niche. Finally, the last step consists in mapping the probability of species occurrence. This method was applied (*i*) to establish the ecological niche (*sensu* Hutchinson [18]) of *L. digitata* (Fig. S2), (*ii*) to model its current spatial distribution and (*iii*) to project its future distribution in the context of climate change, using CMIP5 scenarios. Current spatial distribution of *L. digitata,* measured as probability of occurrence, was assessed for the period 1982–2009 from the ecological niche of the species based on maximum annual SST, bathymetry and annual SSS. We modeled the future potential distribution of *L. digitata* for the 21^st^ century using the NPPEN model with RCP climate scenarios for SST as input. Decadal mean of maximum SST were calculated for each climate model and level of warming (from RCP2.6 to RCP8.5).

#### Selection of the best combination of environmental variables

The selection of the best abiotic variables to model the geographical range of a species is a common issue in ecological niche modeling [38]. We established twelve combinations of environmental factors from the expert knowledge on the ecology of *L. digitata* (Table 1). Temperature and bathymetry are known to be among the most important factors regulating the horizontal and the vertical distribution of the species, respectively. In our model, temperature and bathymetry were thereby the two core variables. We considered mean temperature, both minimum and maximum temperature and maximum temperature alone (Table 1).

**Table 1:** Effects of different combinations of environmental factors on the performance of the model NPPEN applied on *Laminaria digitata.*

Experiments	Bathymetry	Tmean	Tmin	Tmax	Salinity	Substrate	Turbidity	AUC
1.1	x	x						0.65±0.02
1.2	x		x	x				0.63±0.02
1.3	x			x				0.63±0.02
2.1	x	x			x			0.70±0.02
2.2	x		x	x	x			0.72±0.01
2.3	x			x	x			0.82±0.02
3.1	x	x				x		0.67±0.11
3.2	x		x	x		x		0.69±0.08
3.3	x			x		x		0.61±0.04
4.1	x	x					x	0.61±0.04
4.2	x		x	x			x	0.62±0.07
4.3	x			x			x	0.65±0.02

The performance of the model was assessed with the procedure AUC (mean AUC and standard deviation; Methods). Tmean, Tmin and Tmax represent mean, minimum, maximum annual Sea Surface Temperatures, respectively.

Other variables known to influence the presence or the absence of *L. digitata* were considered: salinity, substrate type and turbidity. *L. digitata* can be exposed to major salinity changes during tides cycles [8] but over a long temporal scale, the species exhibit optimal growth for a salinity ranging from 20 to 45 [39]. *L. digitata* is found in the upper sub-littoral fringe in sheltered or moderately exposed sites and exclusively on rocky shore. The macroalga supports high hydrodynamics and prefers clear or slightly turbid waters. The light penetration, which enables the macroalga to perform photosynthesis, is also obviously a key parameter for the growth of the species. This factor is a function of both bathymetry and water turbidity.

We performed twelve simulations (experiments) with different combinations of abiotic variables in order to obtain one set of variables for which the model shows the best ability to reproduce the observed spatial distribution of *L. digitata*. To assess the performance of the models in our twelve experiments, we applied the method AUC (‘Area Under Curve’ of the Receiver Operating Characteristic), which provides a single value representing the model accuracy. AUC plots the false positive rate (1-specificity) against the true positive rate (sensitivity) and calculates the area under the curve. This area (also called “AUC value”) ranges from 0.5 for a random model to 1 for a perfect one.This method does not require the selection of arbitrary thresholds [40]. Although this method was first developed for presence-absence models, absences can be replaced by pseudo-absences, also termed background locations [41], [42]. We used a cross-validation procedure, selecting 70% of data to run the model NPPEN and 30% to evaluate its performance. These background locations were chosen randomly 100 times to provide an average and a standard deviation of the AUC value.

#### Adequacy between AVHRR data and RCP scenarios

Before using SST data from RCP scenarios, we compared the two datasets for the common period 2006–2009 by applying a normalized Taylor’s diagram [43]. The Taylor’s diagram (Fig. S3) allows assessing the performance of models by summarized in a single diagram the standard deviation of the data and those of the model, the Root Mean Square Deviation (RMSD) and the correlation coefficient *R* between the model and the data [44].

## Results and Discussion

### Selection of the Best Combination of Environmental Variables

Table 1 summarizes the analysis for each of the twelve combinations of abiotic variables tested and shows that the run based on bathymetry, maximum annual SST and salinity (experiment 2.3) presents the highest AUC value (AUC = 0.82±0.02). Therefore, we used this triplet of environmental factors to evaluate the present and the future distribution of *L. digitata*. However, it is important to note that the number of factors regulating the observed distribution in nature is much larger. Although here we took into account three parameters, we caution that on local basis, some parameters may be important. Interspecific interaction, the strength of local wind and its effects on wave and currents, sediments type, are some examples of parameters that may locally improve our projections [45], [46], [47].

### Modeling of the Ecological Niche of *L. digitata*

The niche was modeled by applying the model NPPEN on the set of explanatory variables listed below: bathymetry, maximum annual SST and salinity. Probabilities of occurrence higher than p = 0.05 were found at bathymetries between 0 and 12 m (mode between 0 and 5 m), at maximum annual SSTs ranging from 10°C to 20°C (mode around 15°C) and at salinities between 32 and 36 (mode around 34–35), a result in agreement with the ecological knowledge of the species [6], [12], [14] (Fig. S2).

### Spatial Distribution of *L. digitata* for the Period 1982–2009

**Figure 1:**
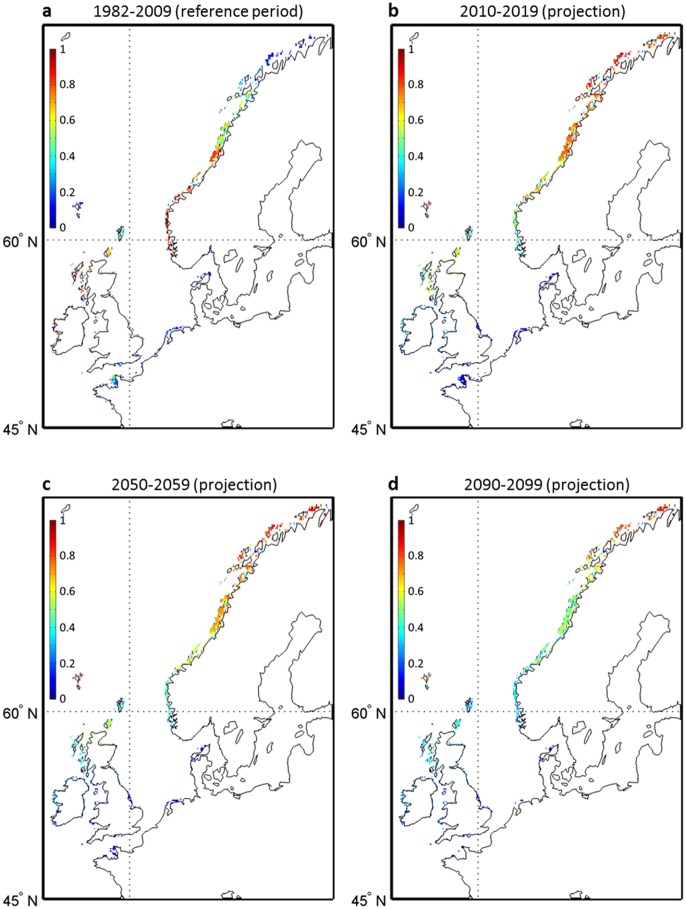
Current and projected spatial distribution in the probability of occurrence of *Laminaria digitata* in the Northeast Atlantic calculated from NPPEN. **a**, mean probability of occurrence for the period 1982–2009. **b–d**, projected changes in the spatial distribution of the seaweed for the decades 2010–2019 (b), 2050–2059 (c) and 2090–2099 (d) based on the average of 6 runs (i.e. two climate models and three RCP scenarios; Methods). Colored white geographical cell denotes a nil probability.

The modeled ecological niche was subsequently projected onto the geographical space to map the current distribution (1982–2009) of *L. digitata* in term of probability of occurrence (Fig. 1a). A high probability of occurrence corresponds to an environment highly suitable for the species. All the southern and northern limits, the sparse distribution along the northern coasts of France, Belgium, Netherlands and Germany and the total absence of the species in the Baltic Sea were well reproduced [9].

### Adequacy between AVHRR Data and RCP Scenarios

Since the present distribution of probability of occurrence was based on AVHRR SST data (1982–2009) and projections of future probabilities on SST data coming from RCP scenarios (2006–2100), we compared both observed and modeled SSTs for the common period 2006–2009 to prevent any bias due to the different SST datasets. This comparison was made by the use of a Taylor’s diagram. All correlation coefficients with AVHRR data were larger than 0.9, which indicate that RCP SSTs were close to AVHRR data for the overlapping period 2006–2009. These analyses suggest that any significant changes between observed- and projected-SST-based probabilities of occurrence are unlikely to be related to the difference between SST datasets (Fig. S3).

### Projections of the Spatial changes in the Distribution of *L. digitata* for the 21^st^ Century

Applying a multi-model and multi-scenarios approach [48], we used SST data originating from the new IPCC Representative Concentration Pathways (RCP) scenarios [25] to evaluate the effect of climate-induced changes in temperatures on both the spatial range and local densities of *L. digitata*. Using two climate models and three RCP scenarios (6 runs), we established projections of the spatial distribution of the kelp for each decade from 2010 to 2099. We first calculated the average probability of occurrence resulting from all runs for each decade to evaluate the potential changes in the both expected abundance and spatial distribution of the kelp until the end of the century (Fig. 1b–d). At the southern edge of the spatial distribution of the species, a substantial decline in the probability of occurrence is expected as early as the 2010s from French to Danish coasts and first nil average probabilities are observed in the 2050s (Fig. 1c). Although a diminution is clearly expected over Scotland, some populations (e.g. northern part of Scotland) are likely to persist until the end of this century (Fig. 1d). Extensive changes are also expected to take place in the southern coastal regions of Norway. A major poleward biogeographic movement of the core region (i.e. region with the highest probabilities) is likely to be observed along the Norwegian coasts in the next decades.

**Figure 2:**
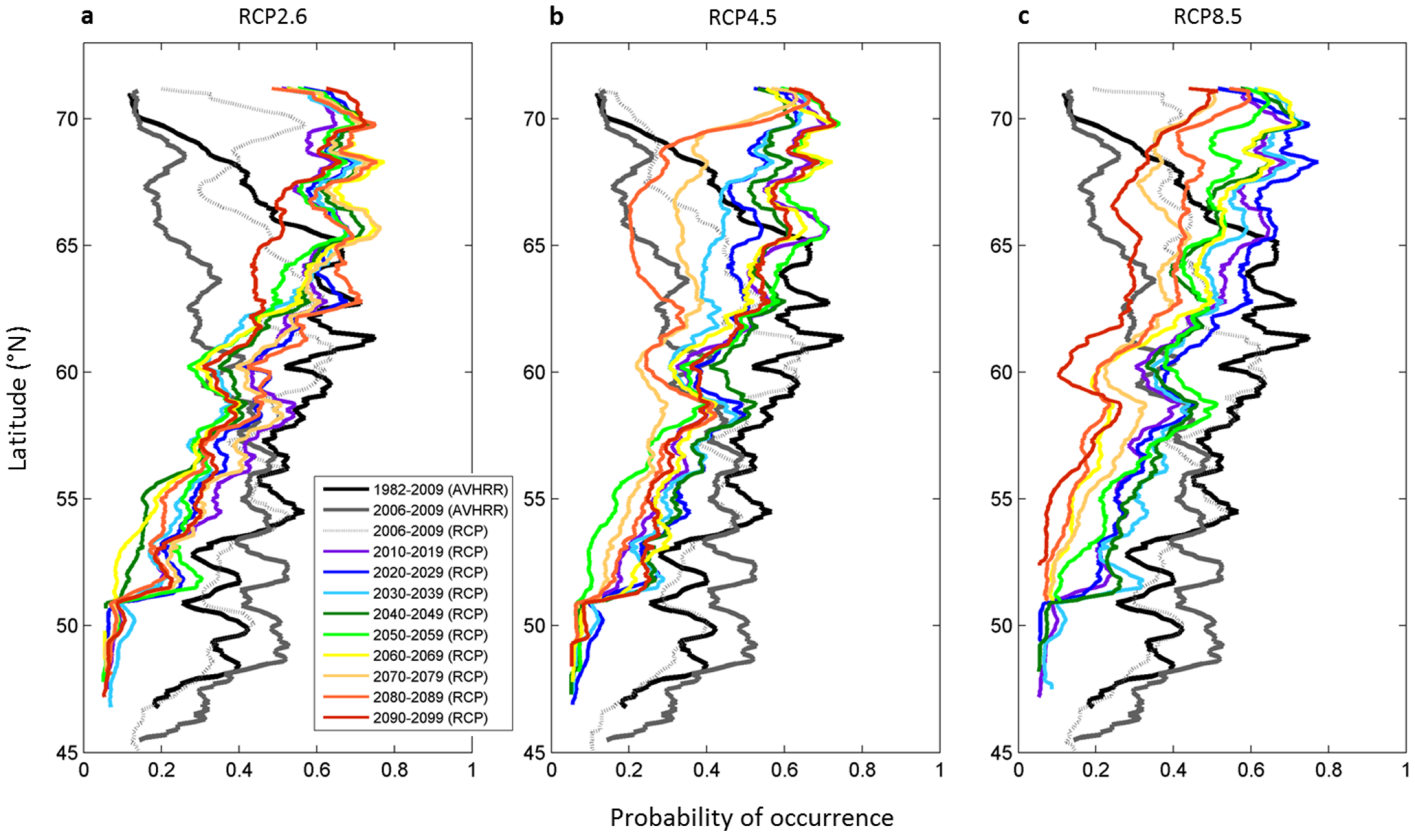
Latitudinal gradient in the probability of occurrence of the kelp *Laminaria digitata* along the European coasts, calculated for the periods 1982–2009 with AVHRR SST data and for 2006–2009 and each decade of the 21^st^ century with RCP-SST data. Projected changes in the probability of occurrence are presented for three RCP scenarios: **a**, the low RCP2.6; **b**, the medium RCP4.5 and **c**, the high RCP8.5.

We examined the latitudinal shifts expected under three levels of warming, taken as a reference the period 1982–2009 (Fig. 2). Note that for the overlapping period 2006–2009, the probabilities calculated from observed (AVHRR) and modeled (RCP SST) data were in general close at latitudes inferior to 62°N (mean probability difference = 0.02). More differences were observed north of 62°N (mean probability difference = 0.15). It is well known that SST derived from satellite radiometric data are better evaluated in temperate areas than under high-latitudes where cloudiness related problems are recurrent [49]. Whatever the intensity of warming, a substantial decline in the probability takes place between 45 and 62°N, as early as in the 2010s. From the decade 2020–2029 to the end of the 21^st^ century, projected changes are subsequently smaller. With the low scenario (RCP2.6), changes taking place after 2019 are in general inferior to 0.2 (Fig. 2a) but the alterations of the latitudinal probabilities become larger when the intensity of global warming increases (Fig. 2b–c). North of 67°N, future probabilities are higher than the present ones for all scenarios.

**Figure 3:**
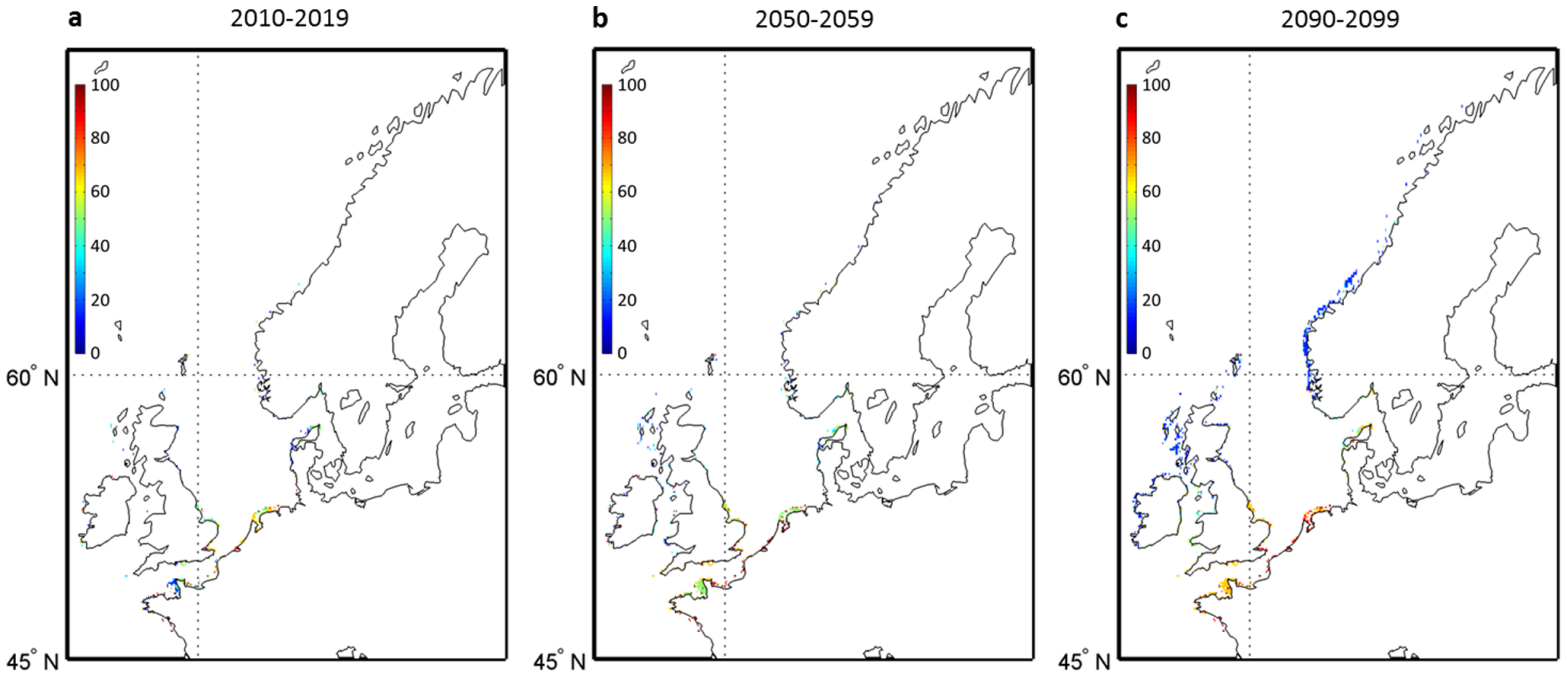
Percentage of models forecasting a disappearance of *L. digitata*. **a,** for the decade 2010–2019; **b,** for the decade 2050–2059 and **c,** for the decade 2090–2099. In each grid cell, a model is considered to predict the disappearance of the species when the probability of occurrence for the reference period (1982–2009) is higher than 0.05 and lower than this threshold for a given decade.

To explore the effects of both uncertainties from the two climate models and the intensity of warming from the three RCP scenarios on the range of occurrence probabilities, we calculated the percentage of runs projecting a disappearance of *L. digitata* during the decades 2010–2019, 2050–2059 and 2090–2099 (Fig. 3). The term ‘disappearance’ is used in geographical cells for which the probability was higher than 0.05 in the reference period 1982–2009 and lower than this threshold for a given decade. For the 2010s, a few projections suggest some local extirpation of *L. digitata* from the French to the Danish coasts and in some areas from the southern parts of England (Fig. 3a) but the percentages remain low and the majority of projections only predict a regression of the populations (Fig. 1b and Fig. 2). However, as early as the decade 2050–2059, between 50 and 100% of the models suggest local extirpations of this species in the previously described regions. At the end of the 21^st^ century (decade 2090–2099), the rate of models predicting a disappearance substantially increases for most regions representing the southern part of the spatial distribution of the species and the areas in which the species is likely to become extinct spread northwards: a few projections (less than 30%) even suggest local extirpation in the southern coastal regions along Norway (Fig. 3c).

### Long-term Changes in the Probability of Occurrence of *Laminaria digitata* in Roscoff

**Figure 4:**
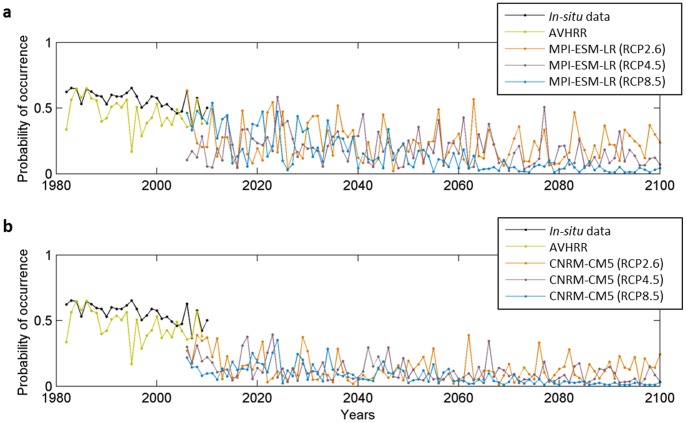
Observed and projected long-term changes in the probability of occurrence of *Laminaria digitata* in Roscoff, calculated from maximum SST at the site “Astan” (Brittany, France - 48° 46′ 40 N, 3° 56′ 15 W). Probabilities are based on maximum Sea Surface Temperature datasets (AVHRR, *in-situ* measurements and RCP scenarios using both CNRM-CM5 and MPI-ESM-LR models), a fixed bathymetry (5 m) and salinity (35.3). **a**, MPI-ESM-LR model and **b**, CNRM-CM5 model.

**Figure 5:**
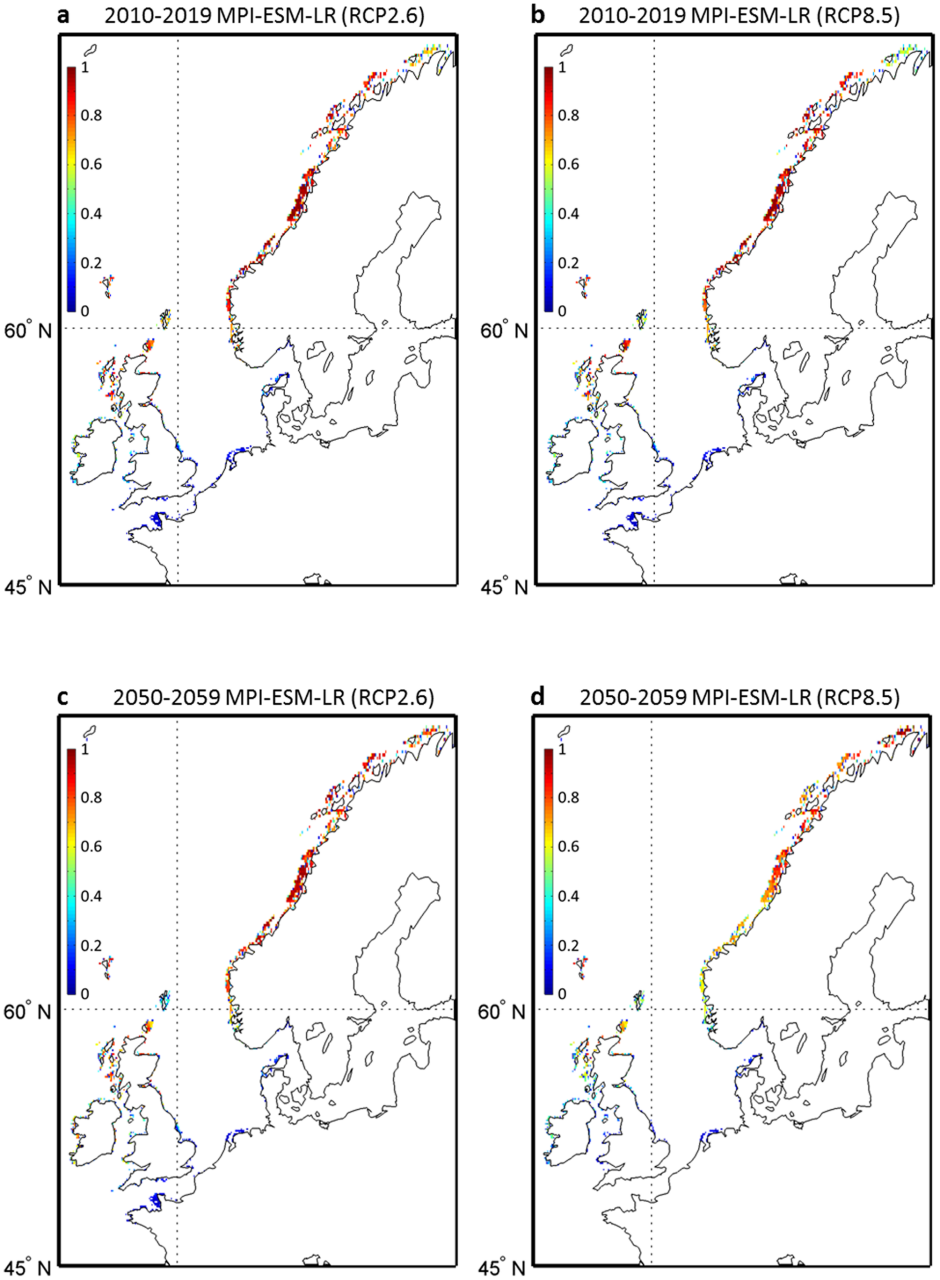
Projections of short-term (2010–2019) and long-term (2050–2059) changes in the spatial distribution of the probability of occurrence of *L. digitata* in Europe calculated from NPPEN model and based on maximum annual SST estimated by the MPI-ESM-LR model. **a,** mean probability of occurrence for the period 2010–2019 based on maximum SST assessed from the scenario RCP2.6. **b,** mean probability of occurrence for the period 2010–2019 based on maximum SST assessed from the scenario RCP8.5. **c,** mean probability of occurrence for the period 2050–2059 based on maximum SST assessed from the scenario RCP2.6. **d,** mean probability of occurrence for the period 2050–2059 based on maximum SST assessed from the scenario RCP8.5. Colored white geographical cell denotes a nil probability. The colorbar indicates the probability of occurrence of *L. digitata*.

Because our projections suggest the likely disappearance of *L. digitata* in some countries (e.g. France) as early as the 2050s, we used local *in-situ* temperature data to calculate the long-term changes in the probability of occurrence of this species in an area where the seaweed is near to the southern edge of its spatial distribution (Roscoff, France) and is being exploited for its alginate and iodine content [7]. Probabilities based on *in-situ* measurements were compared to AVHRR temperature-based and projected probabilities for the overlapping period 2006–2009 (Fig. 4). This analysis showed that MPI-ESM-LR-based probabilities of occurrence were closer to the *in-situ*-based than the probabilities based on CNRM-CM5 model, especially for scenarios RCP4.5 and RCP8.5. A long-term decline in the probability of occurrence is expected to take place in the first half of this century, mainly for scenarios RCP4.5 and RCP8.5. For Scenario RCP2.6, the intra-decadal variability in the probabilities is elevated, especially for the MPI-ESM-LR model. As the CNRM-CM5 model underestimated the probabilities of occurrence based on *in-situ* measurements, we checked if our conclusions hold by considering exclusively the MPI-ESM-LR model (Fig. 5). We excluded from the comparison scenario RCP4.5, which gave lower probabilities. Probabilities of occurrence in the decade 2010–2019 were close to the average probabilities based on the 6 runs (see Fig. 1b). The projection based on the MPI-ESM-LR-RCP8.5 scenario suggests that extirpation of the species along French coast is very likely if global warming is extensive.

### Potential Shortcomings Related to the use of Ecological Niche Models

The ecological niche is a common concept used in ecology but unfortunately, in the history of ecology, it had several definitions [50], [51]. In 1917, Grinnell used this term to designate the “position occupied by a species in both its biological and physical environment” [52]. In 1927, Elton defined the niche as the functional role of a species within the food chain [53]. Odum, in 1953, introduced the notion of the function of organisms within a community instead of the place. Hutchinson, in 1957, defined the ecological niche as the sum of all the environmental factors acting on the organism [18]. The niche is here a n-dimensional hypervolume and represents ‘the range of tolerance of species when several factors are taken simultaneously’ [18]. Here, as in most ENMs, we used the Hutchinson’s definition of the niche.

Ecological Niche Models such as NPPEN are although increasingly used to investigate the potential effect of global climate change on species distributions [23]. These models have already shown their ability to produce accurate prediction of species distributions based on environmental factors [22]. However, it is important to recall that ENMs do not take into account biotic interactions (e.g. predation, competition and mutualism), dispersal mechanisms and life-history strategies of species [54]. Moreover, these models assume no change in the fundamental niche of species [55]. However, rapid changes in physiological rate (especially thermal tolerance) of *L. digitata* can be excluded since the time-scale of this process is largely greater than a century [56], [57].

Our study provides evidence that the habitat of *L. digitata* along the European coasts, which had suitable environmental conditions for the macroalga in the recent past [9] have been altered by an increase in temperatures. Regional extinction in *L. digitata* may occur as early as the first half of this century, although this figure depends upon the magnitude of future warming and its interaction with regional climatic variability. Although local extirpation may arise directly through physiological stress related to elevated temperature [6], it is as likely that the retreat occurs through an unbalance in the biotic interaction between the kelp and other species, and it is plausible that the species disappears through mass mortality related to disease. In addition to global climate change, *L. digitata* is being affected by multiple stressors related to human activities: increased turbidity, sea level rise, introduction of exotic species [58]. Harvesting of this species will potentially lead to the fragmentation of their populations and it is probable that the interactive adverse effects of climate change with more direct anthropogenic stressors accelerate the retreat of the kelp by reducing gene flows and genetic diversity [58]. Several studies showed that the removal of *L. digitata* has often been accompanied by a concomitant increase in *Saccorhiza polyschides*, a pioneer noncommercial kelp species [9], [11], [15], which may have important adverse consequences not only for the associated biodiversity but for goods and services. Our longer-term projections are rather pessimistic for the future of the kelp in the English Channel and the North Sea. Any reduction of the spatial distribution of *L. digitata* will have important ecological consequences since the macroalga enables the establishment of a large number of animals, typically between 32 and 107 [59] and provides spawning, nursery and feeding grounds for many species of both invertebrates and fishes.

## Supporting Information

Figure S1 **Distribution map of the occurrence data points of** ***Laminaria digitata*** **along European coasts from two datasets (OBIS and EcoKelp) and from the examination of the literature.**
(TIF)

Figure S2 **Modeled ecological niche of** ***Laminaria digitata*** **assessed from NPPEN and based on three environmental factors represented by pairs. a,** Maximum annual SST and bathymetry; **b,** Maximum annual SST and salinity; **c,** Bathymetry and salinity. The colorbar indicates the probability of occurrence of *L. digitata*.
(TIF)

Figure S3 **Comparisons between observed and two modeled SSTs (MPI-ESM-LR and CRNM-CM5) for the period 2006–2009 and three climate scenarios (RCP2.6, RCP4.5 and RCP8.5).** The comparison was made by means of Normalized Taylor’s diagrams. The procedure combines into a single diagram both the linear coefficient of correlation and the Root Mean Square Deviation (RMSD) calculated between observed and modeled SSTs, and the standard deviation of each modeled SST normalized by the standard deviation of AVHRR (Advanced Very High Resolution Radiometer) SSTs.
(TIF)

## References

[1] Edwards M, Richardson AJ (2004) Impact of climate change on marine pelagic phenology and trophic mismatch. Nature 430: 881–884. 15318219 10.1038/nature02808

[2] Beaugrand G (2009) Decadal changes in climate and ecosystems in the North Atlantic Ocean and adjacent seas. Deep-Sea Research II 56: 656–673.

[3] Mann KH (1973) Seaweeds: their productivity and strategy for growth. The role of large marine algae in coastal productivity is far more important than has been suspected. Science 182: 975–981. 17833778 10.1126/science.182.4116.975

[4] Wernberg T, Thomsen MS, Tuya F, Kendrick GA, Staehr PA, et al (2010) Decreasing resilience of kelp beds along a latitudinal temperature gradient: potential implications for a warmer future. Ecology letters 13: 685–694. 20412279 10.1111/j.1461-0248.2010.01466.x

[5] Müller R, Laepple T, Bartsch I, Wiencke C (2009) Impact of oceanic warming on the distribution of seaweeds in polar and cold-temperate waters. Botanica Marina 52: 617–638.

[6] Bartsch I, Wiencke C, Bischof K, Buchholz CM, Buck BH, et al (2008) The genus Laminaria sensu lato : recent insights and developments. European Journal of Phycology 43: 1–86.

[7] Davoult D, Engel CR, Arzel P, Knoch D, Laurans M (2011) Environmental factors and commercial harvesting: exploring possible links behind the decline of the kelp Laminaria digitata in Brittany, France. Cahiers de biologie marine 52: 429–434.

[8] Lüning K (1990) Seaweeds: their environment, biogeography, and ecophysiology: Wiley-Interscience.

[9] Birkett D, Maggs C, Dring M, Boaden P, Seed R (1998) Infralittoral reef biotopes with kelp species. Vol VII An Overview of Dynamic and Sensitivity Characteristics for Conservation Management of Marine SACs: 174.

[10] Van den Hoek C, Breeman A, Stam W (1990) The geographical distribution of seaweed species in relation to temperature: present and past. Developments in Hydrobiology 57.

[11] Arzel P (1998) Les laminaires sur les côtes bretonnes: évolution de l’exploitation et de la flottille de pêche, état actuel et perspectives: Editions Quae.

[12] Pérez R (1971) Ecologie, croissance et regeneration, teneurs an acide alginique de Laminaria digitata sur les cotes francaises de la Manche. Revue des Travaux de l’Institut des Pêches Maritimes 35: 287–346.

[13] Cosson J (1973) Influence des conditions de culture sur le développement de Laminaria digitata (L.) Lam.(Phéophycées, Laminariales). Soc Phycol de France Bull: 104–112.

[14] Bolton J, Lüning K (1982) Optimal growth and maximal survival temperatures of Atlantic Laminaria species (Phaeophyta) in culture. Marine Biology 66: 89–94.

[15] Engelen AH, Lévèque L, Destombe C, Valero M (2011) Spatial and temporal patterns of recovery of low intertidal Laminaria digitata after experimental spring and autumn removal. CBM-Cahiers de Biologie Marine 52: 441.

[16] Cosson J (1999) Sur la disparition progressive de *Laminaria digitata* sur les côtes du Calvados (France). Cryptogamie Algologie 20: 35–42.

[17] Gevaert F, Janquin MA, Davoult D (2008) Biometrics in Laminaria digitata: A useful tool to assess biomass, carbon and nitrogen contents. Journal of Sea Research 60: 215–219.

[18] Hutchinson GE (1957) Concluding remarks. Cold Spring Harbor Symposium Quantitative Biology 22: 415–427.

[19] Méléder V, Populus J, Guillaumont B, Perrot T, Mouquet P (2010) Predictive modelling of seabed habitats: case study of subtidal kelp forests on the coast of Brittany, France. Marine Biology 157: 1525–1541.

[20] Bekkby T, Rinde E, Erikstad L, Bakkestuen V (2009) Spatial predictive distribution modelling of the kelp species Laminaria hyperborea. ICES Journal of Marine Science: Journal du Conseil 66: 2106–2115.

[21] Bekkby T, Moy FE (2011) Developing spatial models of sugar kelp (*Saccharina latissima*) potential distribution under natural conditions and areas of its disappearance in Skagerrak. Estuarine, Coastal and Shelf Science.

[22] Beaugrand G, Lenoir S, Ibanez F, Manté C (2011) A new model to assess the probability of occurrence of a species based on presence-only data Marine Ecology Progress Series. 424: 175–190.

[23] Lenoir S, Beaugrand G, Lecuyer E (2011) Modelled spatial distribution of marine fish and projected modifications in the North Atlantic Ocean. Global Change Biology 17: 115–129.

[24] Rombouts I, Beaugrand G, Dauvin J-C (2012) Potential changes in benthic macrofaunal distributions from the English Channel simulated under climate change scenarios. Estuarine, coastal and Shelf Science 99: 153–161.

[25] Moss RH, Edmonds JA, Hibbard KA, Manning MR, Rose SK, et al (2010) The next generation of scenarios for climate change research and assessment. Nature 463: 747–756. 20148028 10.1038/nature08823

[26] Taylor KE, Stouffer RJ, Meehl GA (2012) An overview of CMIP5 and the experiment design. Bulletin of the American Meteorological Society 93: 485.

[27] Gayral P, Cosson J (1973) Exposé synoptique des données biologiques sur la laminaire digitée Laminaria digitata: Organisation des Nations Unies pour l’alimentation et l’agriculture.

[28] Pedersen M, Snoeijs P (2001) Patterns of macroalgal diversity, community composition and long-term changes along the Swedish west coast. Hydrobiologia 459: 83–102.

[29] Billot C, Engel CR, Rousvoal S, Kloareg B, Valero M (2003) Current patterns, habitat discontinuities and population genetic structure: the case of the kelp Laminaria digitata in the English Channel. Marine Ecology Progress Series 253: 21.

[30] Hardy F, Guiry M (2006) A Check-list and Atlas of the Seaweeds of Britain and Ireland (revised ed.), London: British Phycological Society, available from Koeltz Books, Germany, pp. x+435. ISBN 3-906166-35-X.

[31] Casey K, Brandon T, Cornillon P, Evans R (2010) The past, present and future of the AVHRR Pathfinder SST program. Oceanography from space: Revisited.

[32] Smith WHF, Sandwell DT (1997) Global sea floor topography from satellite altimetry and ship depth soundings. Science 277: 1956–1962.

[33] Levitus SE (1982) Climatological atlas of the world ocean. Washington DC: US Government Printing Office.

[34] Acker JG, Leptoukh G (2007) Online analysis enhances use of NASA earth science data. Eos, Transactions American Geophysical Union 88: 14.

[35] Ibañez F (1981) Immediate detection of heterogeneities in continuous multivariate, oceanographic recordings. Application to time series analysis of changes in the bay of Villefranche sur Mer. Limnology and Oceanography 26: 336–349.

[36] Legendre P, Legendre L (1998) Numerical Ecology. Amsterdam: Elsevier Science B.V. 853 p.

[37] Mielke PW, Berry KJ, Brier GW (1981) Application of multiresponse permutation procedures for examining seasonal changes in monthly mean sea-level pressure patterns. Monthly Weather Review 109: 120–126.

[38] Stockwell D (2007) Niche modeling: predictions from statistical distributions: CRC Press.

[39] Karsten U (2007) Research note: Salinity tolerance of Arctic kelps from Spitsbergen. Phycological Research 55: 257–262.

[40] Fielding AH, Bell JF (1997) A review of methods for the assessment of prediction errors in conservation presence/absence models. Environmental Conservation 24: 38–49.

[41] Wiley E, McNyset KM, Peterson AT, Robins CR, Stewart AM (2003) Niche modeling and geographic range predictions in the marine environment using a machine-learning algorithm. Oceanography 16: 120–127.

[42] Philips SJ, Anderson RP, Shapire RE (2006) Maximum entropy modeling of species geographic distributions. Ecological Modelling 190: 231–259.

[43] Taylor KE (2001) Summarizing multiple aspects of model performance in a single diagram. J Geophys Res 106: 7183–7192.

[44] Raybaud V, Nival P, Prieur L (2011) Short time-scale analysis of the NW Mediterranean ecosystem during summer-autumn transition: A 1D modelling approach. Journal of Marine Systems 84: 1–17.

[45] Hawkins S, Hartnoll R (1985) Factors determining the upper limits of intertidal canopy-forming algae. Marine ecology progress series Oldendorf 20: 265–271.

[46] Hawkins SJ, Moore P, Burrows MT, Poloczanska E, Mieszkowska N, et al (2008) Complex interactions in a rapidly changing world: responses of rocky shore communities to recent climate change. Climate Research 37: 123–133.

[47] Hawkins S, Sugden H, Mieszkowska N, Moore P, Poloczanska E, et al (2009) Consequences of climate-driven biodiversity changes for ecosystem functioning of North European rocky shores. Marine Ecology Progress Series 396: 245–259.

[48] Laepple T, Jewson S, Coughlin K (2008) Interannual temperature predictions using the CMIP3 multi-model ensemble mean. Geophys Res Lett 35: L10701.

[49] Podestá GP, Arbelo M, Evans R, Kilpatrick K, Halliwell V, et al (2003) Errors in high-latitude SSTs and other geophysical products linked to NOAA-14 AVHRR channel 4 problems. Geophysical research letters 30: 1548.

[50] Sainsbury KJ (1982). The ecological basis of tropical fisheries management. In: Pauly D and Murphy GI, editors. Theory and management of tropical fisheries: ICLARM Conference Proceedings 9, 167–188.

[51] Whittaker RH, Levin SA, Root RB (1973) Niche, habitat, and ecotope. American Naturalist: 321–338.

[52] Grinnell J (1917) The niche-relationships of the California Thrasher. The Auk 34: 427–433.

[53] Elton C (1927) Animal ecology. London: Sidgwick and Jackson.

[54] Thuiller W (2004) Patterns and uncertainties of species’ range shifts under climate change. Global Change Biology 10: 2020–2027. 10.1111/gcb.12727 PMC4340562 25200514

[55] Peterson AT, Vieglais DA (2001) Predicting species invasions using ecological niche modelling: new approaches from bioinformatics attack a pressing problem. Bioscience 51: 363–371.

[56] Breeman A, Pakker H (1994) Temperature ecotypes in seaweeds: adaptive significance and biogeographic implications. Botanica Marina 37: 171–180.

[57] Clayton M, Wiencke C, Klöser H (1997) New records of temperate and sub-Antarctic marine benthic macroalgae from Antarctica. Polar Biology 17: 141–149.

[58] Valero M, Destombe C, Mauger S, Ribout C, Engel CR, et al (2011) Using genetic tools for sustainable management of kelps: a literature review and the example of Laminaria digitata. Cahiers de biologie marine 52: 467–483.

[59] Lippert H, Iken K, Rachor E, Wiencke C (2001) Macrofauna associated with macroalgae in the Kongsfjord (Spitsbergen). Polar Biology 24: 512–522.

