# Supplementary figures and images for: Decline in Kelp in West Europe and Climate
Source: PLoS One. 2013 Jun 26;8(6):e66044. doi: 10.1371/journal.pone.0066044 (PMC3694085; doi:10.1371/journal.pone.0066044)

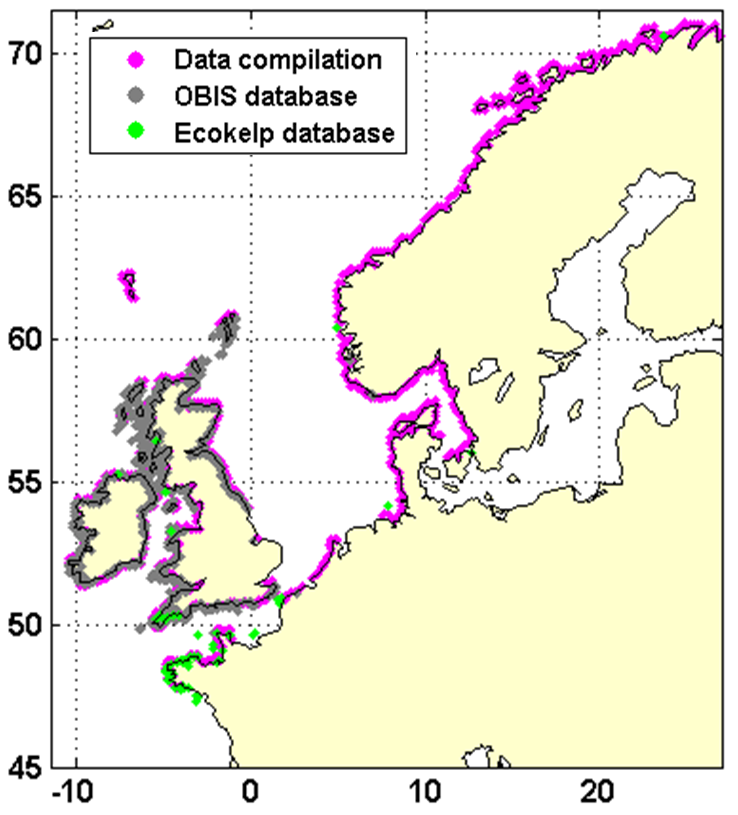

Supplement: Figure S1 — Distribution map of the occurrence data points of Laminaria digitata along European coasts from two datasets (OBIS and EcoKelp) and from the examination of the literature. (TIF) [file pone.0066044.s001.tif]

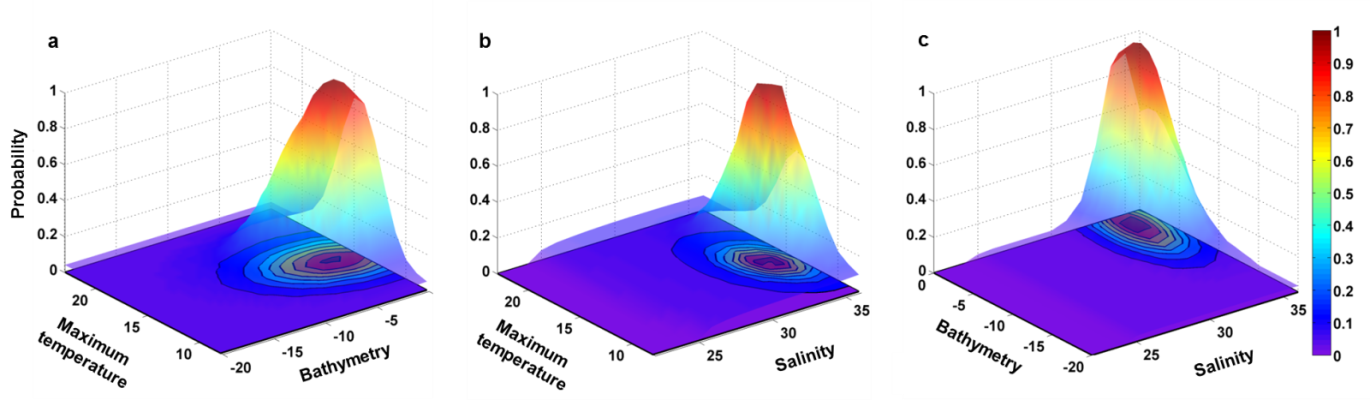

Supplement: Figure S2 — Modeled ecological niche of Laminaria digitata assessed from NPPEN and based on three environmental factors represented by pairs. a, Maximum annual SST and bathymetry; b, Maximum annual SST and salinity; c, Bathymetry and salinity. The colorbar indicates the probability of occurrence of L. digitata. (TIF) [file pone.0066044.s002.tif]

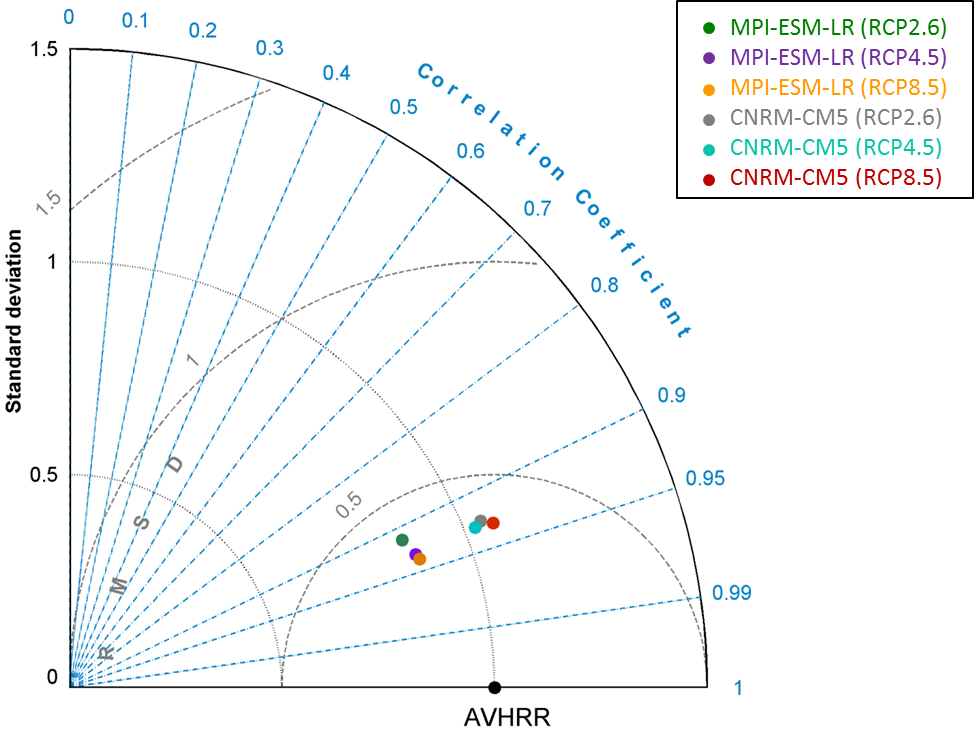

Supplement: Figure S3 — Comparisons between observed and two modeled SSTs (MPI-ESM-LR and CRNM-CM5) for the period 2006–2009 and three climate scenarios (RCP2.6, RCP4.5 and RCP8.5). The comparison was made by means of Normalized Taylor’s diagrams. The procedure combines into a single diagram both the linear coefficient of correlation and the Root Mean Square Deviation (RMSD) calculated between observed and modeled SSTs, and the standard deviation of each modeled SST normalized by the standard deviation of AVHRR (Advanced Very High Resolution Radiometer) SSTs. (TIF) [file pone.0066044.s003.tif]
